# The Goblet Cell Is the Cellular Source of the Anti-Microbial Angiogenin 4 in the Large Intestine Post *Trichuris muris* Infection

**DOI:** 10.1371/journal.pone.0042248

**Published:** 2012-09-06

**Authors:** Ruth A. Forman, Matthew L. deSchoolmeester, Rebecca J. M. Hurst, Steven H. Wright, Alan D. Pemberton, Kathryn J. Else

**Affiliations:** 1 Faculty of Life Sciences, University of Manchester, Manchester, United Kingdom; 2 Division of Veterinary Clinical Sciences, The Roslin Institute, University of Edinburgh, Roslin, United Kingdom; Charité-University Medicine Berlin, Germany

## Abstract

**Background:**

Mouse angiogenin 4 (Ang4) has previously been described as a Paneth cell–derived antimicrobial peptide important in epithelial host defence in the small intestine. However, a source for Ang4 in the large intestine, which is devoid of Paneth cells, has not been defined.

**Methodology/Principal Findings:**

Analysis was performed on Ang4 expression in colonic tissue by qPCR and immunohistochemistry following infection with the large intestine dwelling helminth parasite *Trichuris muris*. This demonstrated an increase in expression of the peptide following infection of resistant BALB/c mice. Further, histological analysis of colonic tissue revealed the cellular source of this Ang4 to be goblet cells. To elucidate the mechanism of Ang4 expression immunohistochemistry and qPCR for Ang4 was performed on colonic tissue from *T. muris* infected mouse mutants. Experiments comparing C3H/HeN and C3H/HeJ mice, which have a natural inactivating mutation of TLR4, revealed that Ang4 expression is TLR4 independent. Subsequent experiments with IL-13 and IL-4 receptor alpha deficient mice demonstrated that goblet cell expression of Ang4 is controlled either directly or indirectly by IL-13.

**Conclusions:**

The cellular source of mouse Ang4 in the colon following *T. muris* infection is the goblet cell and expression is under the control of IL-13.

## Introduction

Human angiogenin (ANG) was originally isolated over 25 years ago as a tumour-derived protein with angiogenic properties [1]. Subsequently, ANG expression was shown not to be limited to neoplastic cells but also expressed by normal epithelial cells, fibroblasts and blood cells [2]. In contrast to humans, who have one Ang gene, mice have six different Ang paralogs (mAng1 to mAng6) (for a review of the functional divergence of the Ang paralogs see [3]).

Mouse angiogenin 4 (Ang4) is encoded for by a gene cluster on chromosome 14 [4]. The angiogenin family contains closely related proteins (72–81% sequence identity) which belong to the RNase superfamily [4]. Although angiogenins were originally implicated in the growth of tumours, subsequent data has demonstrated that not all members of the family are involved in angiogenesis. Indeed, Ang4 has been identified as a Paneth-cell derived anti-microbial peptide important in epithelial host defense against gut-dwelling bacteria in the small intestine [5]. Importantly, the induction of Ang4 by commensal bacteria distinguishes it from other microbicidal proteins such as defensins which do not appear to be regulated by bacteria [5]. Although significant progress has been made in understanding the role of Paneth cell-derived Ang4 in the small intestine, the importance and source of Ang4 in the large intestine is yet to be elucidated.

*Trichuris muris* in the mouse represents a model system, unique amongst GI nematodes. This uniqueness, and ultimate power, lies in the simple differential ability of mouse strains to expel the parasite [6]. Strains of mouse resistant to *T. muris* mount a Th2 response, whereas susceptible mouse strains mount a Th1 response [7]. Resistance is clearly multi-factorial, but appears to be predominantly under the control of IL-13 and STAT-6 via downstream effects on the innate arm of the immune system [8], [9], [10]. Recently, several novel goblet cell-derived factors, regulated by Th2 cytokines, have been proposed as candidates in the immunological rejection of nematodes. These include RELM-beta [11], intelectin [12], [13], [14], [15] and Muc5ac [16]. We have already demonstrated that Ang4 expression correlates with worm expulsion during *T. muris* infection [13], however, the source of Ang4 in the large intestine, which is devoid of Paneth cells, has not been determined.

Here we present data demonstrating that goblet cells are the source of Ang4 in the large intestine. Moreover, Ang4 production occurs independently of TLR4 signalling but is downstream of IL-13 production.

## Results

### Ang4 expression during *T. muris* infection

**Figure 1:**
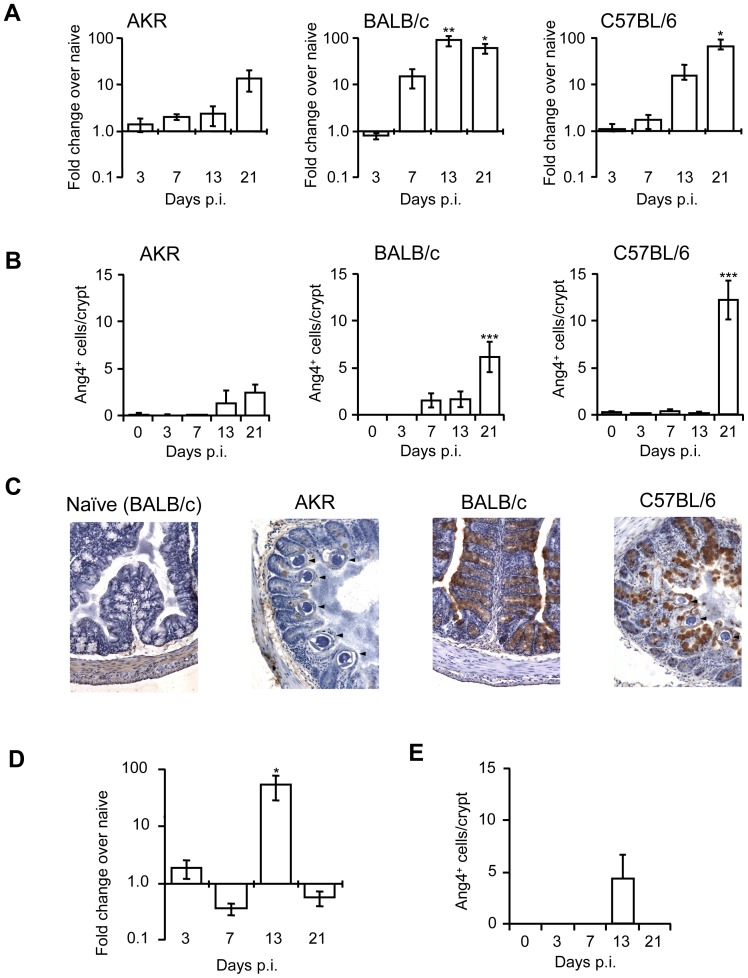
Expression levels of Ang4 in the colons of mice after *T. muris* infection. A; Ang4 mRNA expression was assessed by Q-PCR in samples of colonic tissue from AKR (n = 4–5), BALB/c (n = 4–5) and C57BL/6 mice (n = 4–5) at various times post-infection. B; Quantification of Ang4 protein expression by IHC in colonic tissue from AKR, BALB/c and C57BL/6 mice at various times post-infection. C; examples of immunohistochemical staining for Ang4 in samples of colonic tissue from AKR, BALB/c and C57BL/6 mice at various times post-infection. Original magnification ×200. Arrowheads indicate *T. muris* worm cross-sections in tissue. D; Ang4 mRNA expression was assessed by Q-PCR in samples of colonic tissue from SCID mice (n = 4) at various times post-infection. E; Quantification of Ang4 protein expression by IHC in colonic tissue from SCID mice at various times post-infection. Changes in Ang4 protein levels were confirmed in a second independent experiment in naïve mice and at day 21 post-infection.*p<0.05, **p<0.01, ***p<0.001 One-way ANOVA with post-hoc Dunnett's compared to naïve or d3 control as appropriate.

To confirm previously published microarray data [13], [17] and to investigate the kinetics of Ang4 expression, mice with different expulsion phenotypes were used. BALB/c are resistant to *T. muris* infection with expulsion beginning approximately day 10 p.i. and worms are completely expelled by day 21; this expulsion is associated with a strong Th2 response. C57BL/6 exhibit an intermediate phenotype and expel the worms more slowly exhibiting a reduction in worm burden by day 21 p.i. and most individual mice have cleared their infection by day 35 p.i.. This expulsion kinetic is associated with a mixed Th1 and Th2 response. AKR mice are susceptible and are unable to clear the infection harbouring adult worms at day 35 p.i.; this susceptibility is associated with a dominant Th1 response [18]. Figure 1A shows the level of *Ang4* expression as measured by Q-PCR. In BALB/c mice, levels were increased over 10-fold by day 7 p.i. and reached a significant 94-fold increase by day 13 p.i. In C57BL/6 mice, *Ang4* expression did not increase by 10-fold until day 13 p.i. and peaked at a significant 66-fold increase at day 21 p.i. In contrast, expression levels in AKR mice remained relatively low until day 21 p.i. when they peaked at over 13-fold, although this increase was not significant. Comparing between strains, BALB/c expression levels were significantly higher than AKR and C57Bl/6 at day 13 (p<0.01 and p<0.05 respectively) and AKR alone at day 21 (p<0.05). By day 21 p.i. levels in C57Bl/6 and BALB/c mice were equivalent (p>0.05). This pattern of expression was confirmed by immunohistochemical staining for Ang4 protein (Figure 1B and C). Expression of Ang4 protein appears earlier in BALB/c mice, although it ultimately reaches a higher level in C57BL/6. Levels in AKR mice remained relatively low even at day 21 p.i. and were significantly lower than those in C57Bl/6 mice at day 21 (p<0.01). AKR levels at day 21 were also lower than BALB/C levels at this time-point, although this failed to reach statistical significance (p = 0.0893).

### Control of colonic Ang4 expression during *T. muris* infection

To investigate the mechanism by which Ang4 expression is induced in the large intestine during *T. muris* infection, we initially utilised the SCID mouse (BALB/c background). As V(D)J recombination does not occur, this strain is unable to mount an adaptive immune response and are unable to clear the infection, harbouring adult worms at day 35 post infection. Figure 1 shows the level of Ang4 detectable at the message (D) and protein level (E) in SCID mice. Gene expression was generally low and only significantly increased at day 13 p.i. when it was expressed in two of four mice (Fig. 1D). This result was mirrored by the IHC staining for Ang4 protein, which showed a small increase in cells staining positive for Ang4 at day 13 p.i. (Fig. 1E). Indeed, at day 13 p.i. neither the expression or protein levels of Ang4 in SCID mice were significantly different to those in BALB/c mice (Fig. 1A and B) (p>0.05 for both).

**Figure 2:**
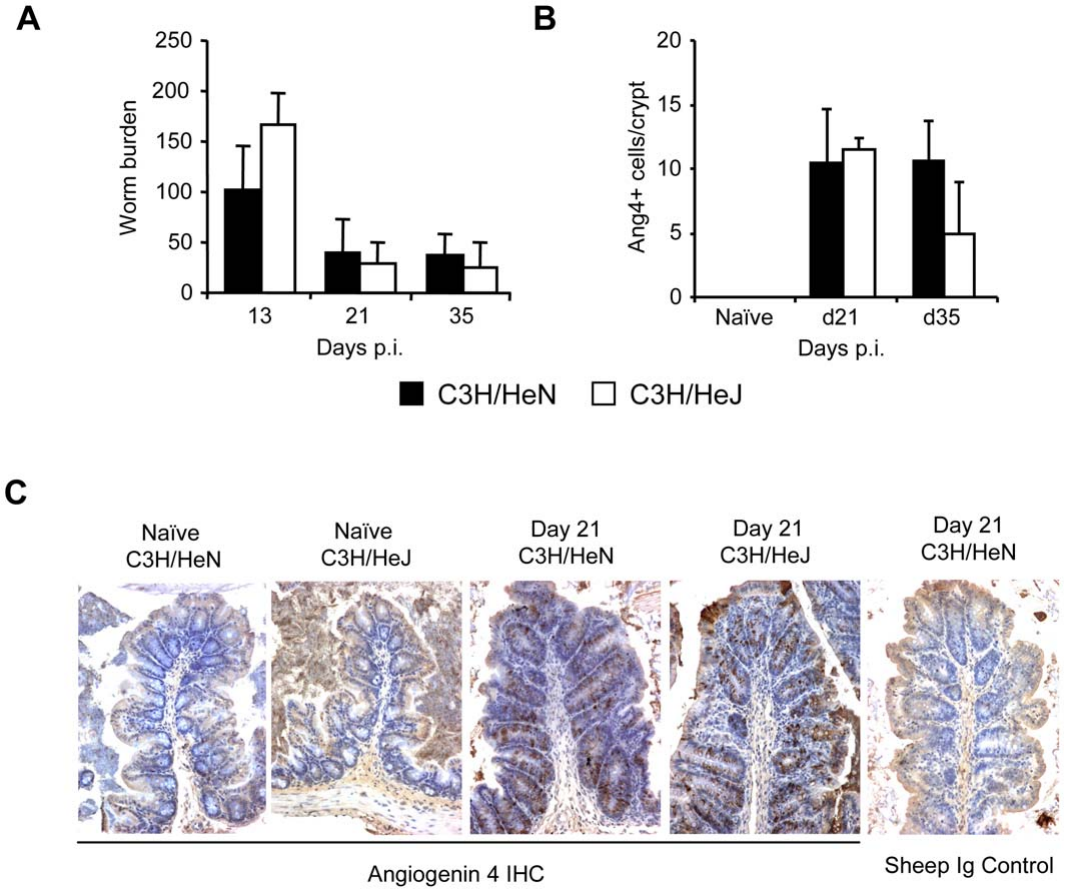
Involvement of pattern recognition receptors in Ang4 expression *in vivo*. A; Worm burden in C3H/HeN and C3H/HeJ mice (n = 4–5) at various times post-infection. B; Quantification of Ang4 protein expression by IHC in colonic tissue from C3H/HeN and C3H/HeJ mice at various times post-infection. IHC results were confirmed in a second independent experiment at selected time points post-infection. C; Ang4 immunohistochemical (IHC) staining in colonic tissue from naïve and *T. muris*-infected C3H/HeN and C3H/HeJ mice, at day 21 p.i. A sheep Ig control for IHC on d21 p.i. C3H/HeN colon tissue was also performed. Original magnification ×200.

We next investigated the involvement of the pattern recognition receptor, TLR4, in the expression of Ang4. C3H/HeN mice respond as normal to ligand binding, whereas C3H/HeJ mice have a natural inactivating mutation of TLR4. TLR4 has been shown not to be essential for expulsion of *T. muris* [19] and few worms remained in either C3H/HeJ or C3H/HeN mice at days 21 and 35 p.i. following a high-dose infection (Fig. 2A). Importantly, similar levels of Ang4 expression were seen in TLR4 wild-type and deficient mice indicating that signalling via TLR4 is not important in the expression of Ang4 (Fig. 2B and C).

**Figure 3:**
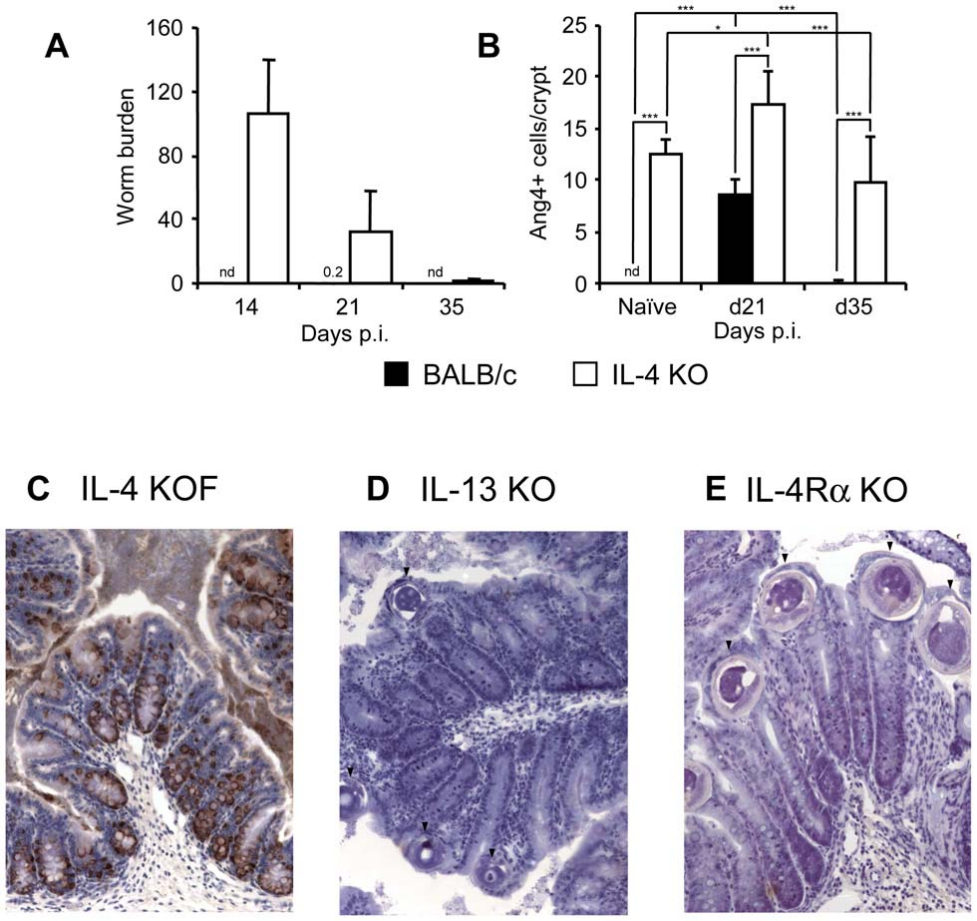
Involvement of IL-4 and IL-13 in Ang4 expression *in vivo*. A; Worm burden in female IL-4 KO (BALB/c background) (n = 5 for naïve mice and n = 7–8 for infected mice) and BALB/c (n = 4 for naïve mice and n = 5–6 for infected mice) mice at various times p.i. 0.2 represents 1 worm present in 1 BALB/c mouse at d21 p.i.. B; Quantification of Ang4 protein expression by IHC in colonic tissue from female IL-4 KO and BALB/c mice at various times p.i.. Figures represent combined data from two experiments. C; Examples of IHC staining for Ang4 in samples of colonic tissue from female IL-4 KO mice, D; IL-13 KO mice (n = 7) and E; IL-4Rα KO mice (n = 5) at 21 days p.i. Nd = none detected. Arrowheads indicates *T. muris* worm cross-sections in tissue. *p<0.05, **p<0.01, ***p<0.001 One-way ANOVA with post-hoc Tukey's analysis.

As early, high level expression of Ang4 only occurred in mice mounting a Th2-dominated immune response, we investigated the need for IL-4 and IL-13 in Ang4 production by analysing levels of Ang4 protein in IL-4 KO, IL-13 KO and IL-4Rα KO. Female IL-4 KO mice on a BALB/c background maintain resistance to *T. muris* infection by compensating with IL-13 to initiate a Th2 immune response [20]. This allows the importance of IL-4 in Ang4 production to be assessed during the expulsion process. Figure 3 shows that female IL-4 KO mice expelled *T. muris* but with slower kinetics than the BALB/c mice which expelled by day 14 p.i. except for 1 mouse which harboured 1 worm at day 21 p.i. (A). Infection of all mice was confirmed by serology (data not shown). Importantly, female IL-4 KO mice produced Ang4 (B and C) with Ang4 expressing cells present in naïve IL-4 KO mice and Ang4 expression remained significantly higher than wildtype mice through the course of infection. Following infection both BALB/c and IL-4KO mice demonstrate an increase in Ang4 with a return to baseline levels following worm expulsion. In the absence of IL-13 (IL-13 KO mice, Fig. 3D) or IL-13 and IL-4 signalling (IL-4Rα KO mice, Fig. 3E), *T. muris* was not expelled (presence of worms visible in histology sections (Fig. 3D and E)) and Ang4 was not expressed at any time p.i. Indeed, no Ang4-positive goblet cells were detected in the IL-13 KO mice (n = 7) or IL-4Rα KO mice (n = 5). This data suggests that Ang4 is optimally expressed by goblet cells in the large intestine in the presence of both IL-4 and IL-13 but that IL-4 is not essential for its expression.

### Cellular source of Ang4 in the colon

**Figure 4:**
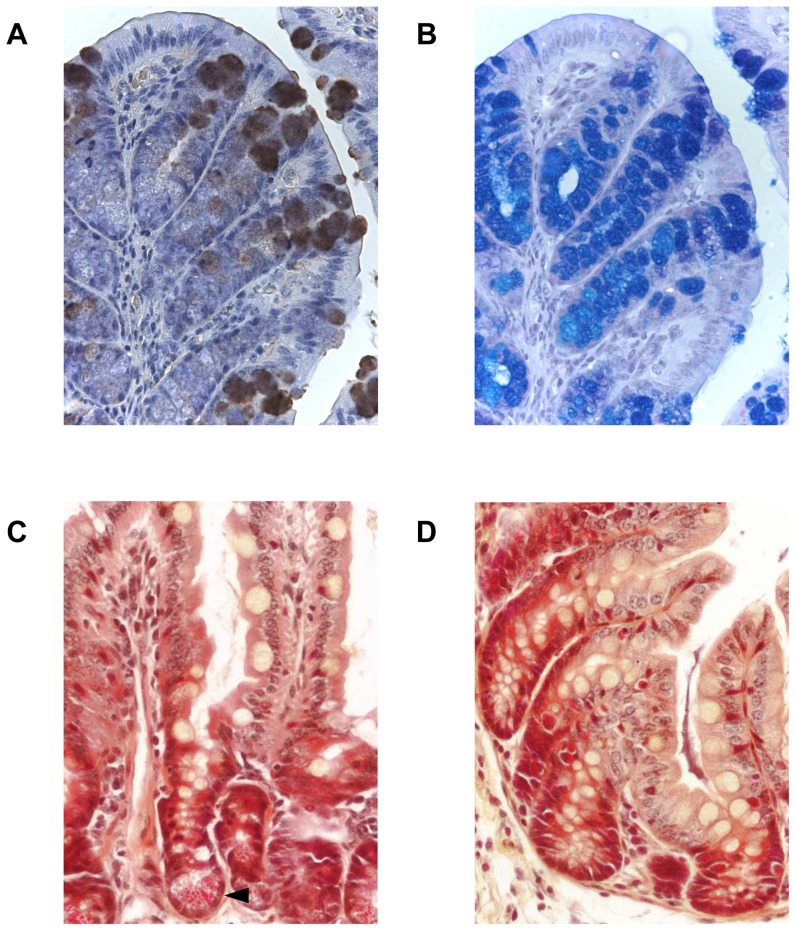
Presence of Ang4, goblet cells and Paneth cell granules in the small and large intestine. A; Ang4 immunohistochemical staining in colonic tissue from C57Bl/6 mice, original magnification ×400. B; Periodic acid/Schiff staining for goblet cells in a serial colonic tissue section from C57Bl/6 mice, original magnification ×400. Philoxine B/tartrazine staining for Paneth cell granules in naïve small intestine (C), and large intestine d21 p.i. with *T. muris* (D). Arrowhead indicates Paneth cell granules, original magnification ×400.

The appearance of the IHC staining suggested the goblet cell was the source of Ang4 expression in the colon and Figure 4 (A and B) shows that the IHC staining overlaps with periodic acid/Schiff staining for mucins. To further investigate the cellular source of Ang4, phloxine B and tartrazine staining was used. This has been shown to stain granules in Paneth cells, the source of Ang4 in the small intestine [21]. Figure 4 shows Paneth cell granule staining in the small intestine in the base of the crypts (Fig. 4C), however, Paneth cells did not appear to be present in the base of the crypts of the large intestine of the in-bred strains of mouse used in this study (Fig. 4D), making them unlikely to be the cellular source of Ang4 post-Trichuris infection. This suggests that goblet cells are the source of Paneth cell-like granules in the inflamed large intestine.

## Discussion

Ang4 is a novel anti-microbial peptide which has previously been implicated in resistance to gut bacteria and is localised to Paneth cells in the small intestine [5]. We have shown, for the first time, that in the absence of Paneth cells, goblet cells are the cellular source of Ang4 in the large intestine. In recent years a lot of research has focused on the importance of Paneth-cell derived antimicrobial products (for review see [22]). Importantly it has been observed that Paneth cell perturbation plays a role in susceptibility to intestinal infection and Crohn's disease [23]. Our novel finding, identifying the goblet cell as the cellular source of Ang4 in the large intestine, suggests that we need to not only focus on the Paneth cell, but also the role of goblet-cell derived anti-microbials.

In addition, we have demonstrated that TLR4 is not necessary for the increase in Ang4 post-infection with *T. muris*. This is in contrast to other anti-microbials which have been demonstrated to be regulated by toll-like receptors. For example, LPS can induce Paneth cell degranulation and production of cryptidins [24]. Nevertheless, this TLR4-independent pathway of goblet cell derived Ang4 production is not surprisingly in light of recent data which has demonstrated that TLR9 and TLR3 agonists are more important for Paneth cell degranulation than TLR4 and TLR5 [25]. Indeed, oral administration of the TLR4 agonist LPS did not induce degranulation until 8 hours after treatment [26] Moreover, Paneth cells have been shown to express TLR9 but not TLR4 and, therefore, any response to LPS cannot be due to a direct action of LPS on the Paneth cells [26]. To our knowledge the toll like receptor expression pattern of goblet cells is unknown, however, it is not unfeasible that a similar receptor repertoire might be present as in Paneth cells.

Resistance to *T. muris* is Th-2 dependent [7], [9], [26]. Female IL-4 KO mice on a BALB/c background are resistant to *T. muris* and this resistance depends on IL-13 [20]. This model therefore provides a means for separating out the relative importance of IL-4 and IL-13 in Ang4 expression in a situation where worms are still expelled. Our data demonstrates that goblet cell expression of Ang4 is controlled, either directly or indirectly, by IL-13 and that IL-4 plays a redundant role as Ang4 expression is seen in the absence of IL-4. Indeed, Ang4 expression is elevated in naïve IL-4KO female mice compared to BALB/c controls, which is possibly due to a compensatory role of IL-13. This supports previously reported data which demonstrated IL-9 induces Ang4 in small intestine Paneth cells in an IL-13 dependant manner [21]. The IL-13-dependant control of Ang4 production also explains the association between Ang4 expression and *T. muris* expulsion. Indeed, the increased Ang4 expression is most likely to reflect the IL-13 dependent control of worm expulsion; rather than implying Ang4 as an anti-parasitic molecule. Nevertheless, goblet-cell derived products are known to be important during *T. muris* infection, as demonstrated by Muc5ac deficient mice which are completely susceptible to infection [16]. The low but significant increase in Ang4 expression seen in goblet cells of SCID mice post-infection points to the presence of innate cellular sources of IL-13. Indeed, NK cells have been shown to make IL-13 in the context of *T. muris* infection [27]. Equally, low numbers of Ang4 positive goblet cells were found in susceptible Th1-dominated AKR mice which are known to still produce low levels of IL-13. Only when IL-13 or IL-13 signalling was removed, in IL-13 KO and IL-4Rα KO mice respectively, did we observe a complete lack of Ang4 positive goblet cells.

In conclusion, our study identifies the goblet cell as the cellular source of angiogenin 4 in the large intestine. We also demonstrate that the expression of Ang4 by goblet cells in the large intestine is independent of TLR4 and IL-4 but dependent on IL-13.

## Materials and Methods

### Animals, *T. muris* and E/S protein

AKR, BALB/c, C57BL/6, C3H/HeN and C3H/HeJ mice were purchased from Harlan U.K. (Bicester, U.K.). SCID, IL-4-, IL-4Rα- and IL-13-deficient mice (all BALB/c background) were bred in-house. Male mice were used except in the case of IL-4 deficient mice when females were used. Mice were infected with approximately 150 infective *T. muris* eggs when 8–10 weeks old and sacrificed at various time points after infection and the worm burden in the large intestine assessed as previously described [26]. All animal experiments were performed under the regulation of the Home Office Scientific Procedures Act (1986) and the Home Office approved grant 40/3217. The maintenance of *T. muris*, the method of infection and production of E/S protein were as previously described [28]. E/S was tested for endotoxin content prior to use for in vitro cell stimulation by the chromogenic Limulus amoebocyte lysate assay (Charles River, Charleston, SC) and found to contain lower levels than normal cell culture media.

### Extraction of total RNA, reverse transcription and quantitative PCR

Tissue samples from the junction of the caecum and colon were placed in TRIsure (Bioline, London, UK) and snap frozen in liquid nitrogen. Samples were homogenised using a FastPrep 24 and lysing matrix D (MP Biomedicals, Illkirch, France) and total RNA extracted according to the manufacturer's instructions for TRIsure. Integrity of RNA was confirmed by visualization of the 18S and 28S ribosomal bands under UV light following separation on a 1.5% agarose gel. The concentration of total RNA was measured by absorbance at 260 nm on a Nanodrop ND-1000 spectrophotometer (Labtech International, East Sussex, U.K.). Contaminating genomic DNA was removed by treatment with RNase-free DNase (Promega, Southampton, UK). Total RNA (1.0 µg) was reverse transcribed using BioScript (Bioline) in a final volume of 40 µl according to the manufacturer's instructions and stored at −20°C until used. Quantitative PCR was performed using SYBR green (New England Biolabs, Hitchin, U.K.) on an OPTICON DNA engine with OPTICON Monitor software version 2.03 (Real-Time systems; MJ Research). Amplification of mRNA encoding Hprt1, 18S and β-actin was performed to control for the starting amount of cDNA. Expression levels of genes of interest are shown as fold change over that seen in naïve animals after normalisation to housekeeping gene levels using the ΔΔC^t^ method. Primers used were: GTAATGATCAGTCAACGGGGGAC and CCAGCAAGCTTGCAACCTTAACCA for *Hprt1*, AGTCCCTGCCTTTGTACACA and GATCCGAGGGCCTCACTAAC for *18S*, GTGGGCCGCTCTAGGCACCAA and CTCTTTGATGTCACGCACGATTTC for *β-actin* and CTCTGGCTCAGAATGTAAGGTACGA and GAAATCTTTAAAGGCTCGGTACCC for *Ang4*. All sequences are 5′-3′ with the sense primer given first.

### Histology

Histological sections were prepared from proximal colon tissue fixed in 10% neutral buffered formalin containing 0.9% sodium chloride, 2% glacial acetic acid and 0.05% alkyltrimethyl-ammonium bromide before being embedded in paraffin. 6-µm sections were cut using a microtome and placed on gelatin-coated slides. Prior to all staining, sections were dewaxed with citroclear (HD supplies, Aylesbury, UK) and taken to water through decreasing concentrations of ethanol.

Immunohistochemical staining for Ang4 was performed as follows. Endogenous peroxidase was quenched by incubation with 1.5 U/ml glucose oxidase (Sigma, Gillingham, Dorset, U.K.) in the presence of 1.8 mg/ml glucose and 0.064 mg/ml sodium azide for 20 minutes at 37°C. Antigen retrieval was performed by incubation with pepsin digest-all™ 3 (Zymed Laboratories) for 10 minutes at 37°C. Non-specific binding was blocked with 1.5% donkey serum (Vector Laboratories) and endogenous avidin and biotin binding sites were blocked using a kit according to the manufacturer's instructions (Vector Laboratories). Sections were then incubated with sheep anti-mouse polyclonal Ang4 antibody (12.5 µg/ml) or sheep Ig control in phosphate-buffered saline containing 1.5% donkey serum, followed by F(ab′)_2_ donkey anti-sheep biotin (0.85 µg/ml) (Stratech Scientific, Newmarket, Suffolk, U.K.). After incubation with ABC (avidin-biotin complex) (Vector Laboratories), 3,3′ Diaminobenzidine (DAB; AdB Serotech, Kidlington, Oxon., U.K.) was added and colour development monitored. Sections were counter-stained in HaemQS (Vector Laboratories) for 1 minute, and mounted in aquamount (BDH, Lutterworth, UK).

Mucin staining was by sequential incubation with 1% alcian blue (Sigma) in 3% acetic acid, 1% periodic acid (Sigma), Schiff's reagent (Vicker's laboratories, Pudsey, West Yorkshire, U.K.) and Mayer's haematoxylin (Sigma). Sections were then dehydrated, cleared and mounted in DPX mounting medium (Raymond A Lamb, Eastbourne, UK). For enumeration of immunohistochemical and goblet cell staining, the average number of positive cells from a minimum of 20 crypts was taken from three different sections per mouse.

Paneth cell staining was performed by staining with Mayer's haematoxylin followed by 0.5% Phloxine B. Slides were blotted dry, dipped in 2-ethoxyethanol then in tartrazine-saturated 2-ethoxyethanol, to ensure the absence of water, before staining in fresh tartrazine-saturated 2-ethoxyethanol. Slides were rinsed in 100% ethanol, cleared and mounted in DPX mounting medium.

### Statistical analysis

Statistical analysis was performed using the Student's t test or ANOVA with post-hoc Dunnett's test compared to appropriate control group or post-hoc Tukey's test, as appropriate, with the statistical package GraphPad Prism (GraphPad Software, San Diego, U.S.A.). A probability value of <0.05 was considered significant (*p<0.05, **p<0.01, ***p<0.001).
